# Laser Vision Correction on Patients with Sick Optic Nerve: A Case Report

**DOI:** 10.1155/2011/796463

**Published:** 2011-12-29

**Authors:** Ming Chen

**Affiliations:** Division of Ophthalmology, Department of Surgery, University of Hawaii, 55 S. Kukui Street C-109, Honolulu, HI 96813, USA

## Abstract

This 48-year-old white female with chronic optic disc edema is reported for the discussion of the management for the laser vision correction. Two procedures were considered, one was PRK (photorefractive keratectomy) and the other was LASIK (laser-assisted in-situ keratomileusis). A search strategy was developed to search evidence in the literature to support the decision in the selection of the better procedure for this patient. The evidences also were rigorously appraised for the validity. PRK was selected and performed on the patient with good outcome.

## 1. Introduction

Patients who have decided to have refractive laser surgery need to make a decision on whether to choose LASIK (laser-assisted in-situ keratomileusis) or PRK (photorefractive keratectomy). Additionally, surgeons have the responsibility to suggest which procedure would be the better choice for patients. Laser vision correction procedures have been widely performed in the United States for more than 10 years. As any medical or surgical procedure mature, the nature of suitable candidate changes for various reasons. Some involve the physiologic changes that occur (e.g., neuropathy), quality of vision (e.g., halos), improvement in technology (i.e., femtolaser and wavefront-guided treatments), and clinical studies (e.g., autoimmune disease and thin corneas). Many questions remain unanswered. 

The LASIK procedure involves two steps: the creation of a thin flap of cornea and laser ablation. In creating a flap, a suction device is placed on the eye to stabilize it for creation of the flap. This induces a rise in intraocular pressure.

This case study, concerning the LASIK procedure, will search evidence through the literature regarding the affect of induced pressure to the eye ball with a sick optic nerve. The alternative procedure such as PRK (without induced pressure) deserves detailed research for evidence that may support its efficacy.

## 2. Case Presentation

SM. A 48-year-old white female presented for evaluation of possible laser vision correction.

Descriptions of her eye findings: 20/20 best corrected vision, normal intraocular pressure, simple myopia, normal cornea thickness, normal pupil size, and normal topography. The significant ocular findings: chronic bilateral optic disk edema for ten years.

In the consideration for the selection of either PRK or LASIK as the procedure for this patient, it is necessary to search relevant evidences to support the decision.

PRK and LASIK are laser eye surgery procedures intended to correct a person's vision to reduce dependency on glasses or contacts.

In PRK, the surgeon removes the outer layer of epithelium with various techniques, then an excimer laser ablates and reshapes corneal tissue. Anesthetic drops are used to reduce pain. LASIK uses a microkeratome device or a femtosecond laser under suctioning to increase intraocular pressure to about 65 mmHg up to 100 mmHg for 45 second to cut epithelium and stroma to a thickness of 100–180 microns, and then, an excimer laser ablates corneal tissue under the epithelial/corneal tissue flap [1].

PRK is an excellent alternative when LASIK is not an option. It is suitable for those with less corneal tissue, because it can preserve more corneal tissue. There will be no complications of stromal flap in PRK. Unlike LASIK, it does not require pressure suction on the eye ball. Nevertheless, the drawbacks are more discomfort than LASIK in the first 24 hours after procedure, it requires more healing time than LASIK, trauma may cause complications after surgery, and long-term outcomes are not as well established as older corrective procedures such as LASIK [1].

LASIK is appropriate for people who have more corneal tissue. The benefits are less discomfort than PRK, almost no pain, 20/20 vision or better is typically achieved by the first day, corneal haze is very rare, immediate clear vision, and follow-up enhancements are easier if needed. However, the drawbacks are contraindicated with thinner corneas, flap may dislodge with trauma, increases higher-order aberrations (HOA), nonsmooth flap may lead to astigmatism, flap may result in scars, and postoperational treatment is needed in approximately 5% of patients [1].

A search strategy was developed to search evidence in the literature to support the decision in the selection of the better procedure for this patient.

### 2.1. Search Strategy: Using *PICO*

**PICO:**

Population: patients have sick optic nerve undergoing refractive laser surgery.
Intervention: PRK.
Comparison: LASIK.
Outcome: no damage to optic nerve after procedure.
Boolean operators are the using of combining words and methods to search in electronic databases. The combining words used here are AND, OR.
According to Boolean operators:
AND—will produce references that include both keywords.
OR—will produce references that contain at least one of the keywords
(#1) refractive laser surgery
(#2) PRK
(#3) LASIK
(#4) optic disc
(#5) #1, and #4 include refractive laser surgery and optic disc
(#6) #2, and #4 include PRK and optic disc
(#7) #3, and #4 include LASIK and optic disc
(#8) #2, and #5 include PRK and refractive laser surgery and optic disc
(#9) #3, and #5 include LASIK and refractive laser surgery and optic disc
(#10) randomized control trial
(#11) systematic reviews
(#12) qualitative studies
(#13) #9, #8, and #12
(#14) #9, #8, and #10
(#15) #9, #8, and #11.

Limits: English, research.

The search was conducted over Pubmed, Medline and Cochrane Library. 1492 articles were found, and 5 papers were selected for this case report. Pubmed had 83 articles, and none was selectedfrom Cochrane library review.

### 2.2. Search Result

There were three observational case reports and two experimental studies (see Tables 1 and 2).

LASIK-induced optic neuropathy by Cameron et al. [2] clearly described the case of a subject with a previously normal optic nerve who developed bilateral optic neuropathy after LASIK surgery [2]. The neuropathy manifested with visual field defects. Valid test such as complete eye exam with detailed evaluation of the optic nerve, detail medical history, stereodisc photographs, GDx nerve fiber analyzer, was used rigorously to confirm the diagnosis associated with LASIK pre-op and post-op. Other possible causes of optic neuropathy were also carefully ruled out. The author concluded that optic nerve neuropathy is a dangerous complication of LASIK. It may be due to barotrauma or ischemia from elevation of intraocular pressure by the suction ring during the LASIK procedure. Lee et al. and Montezuma et al. also reported similar cases with the same conclusion [3, 4]. Lee et al. even reported two cases that experienced chronic disc edema pre-op similar to this case pre-op [3]. The two cases he reported had LASIK and suffered acute visual loss. Nevertheless, the experimental parallel control trial of Lester demonstrated no statistically significantly different measurement between before and after a compression of 100 mmHg to the eye for 45 second (as in suction ring pressure of LASIK procedure) in the mean RNFLT (retina nerve fiber layer thickness). This quantitative study showed that the acute increase in intraocular pressure during LASIK did not significantly change the thickness of retina nerve fiber layer in normal eyes [5]. Furthermore, Hamada conducted a prospective consecutive study of 53 normal eyes to compare the RNFLT before LASIK and after LASIK. He concluded that transient extreme elevation of intraocular pressure (IOP) during LASIK does not affect RNFLT in normal myopic eyes for at least one year after surgery [6].

### 2.3. To Critically Evaluate the Evidences Related to the Case

There were two case reports and one case series. Although these are poor-quality studies and rank near the bottom of hierarchy of evidence, these cases provide valuable insights and warning for potential sight-threatening conditions.

The two experimental studies were appraised according to the CASP framework.

1. Both studies have a clear focus:
  P: patients undergoing refractive laser surgery
  I: LASIK (will receive 100 mmHg of pressure for 45 seconds during the procedure) or 100 mmHg of pressure for 45 seconds.
  O: change in RNFLT (retina nerve fiber layer thickness).
2. These were not randomized trials but were prospective consecutive cases.
3. Participants were appropriately allocated to intervention. The groups appeared equivalent (all normal eyes) and had low risk of selection bias with strong internal validity.
4. It was not possible to “blind” due the nature of intervention. However, the outcome measures are objective that will reduce the threat to internal validity.
5. All the participants in these two studies were accounted for at the conclusion. The follow-up rates were 100% that increase both the internal and external validity.
6. The data collection and the follow-up were done in the same way. (This increases internal validity.)
7. The power calculations were not mentioned in the either of the two studies. It is a decrease of external validity. However, Hamada's [6].Study may have enough power with 53 participants for a single outcome measurement.
8. The result showed no statistically difference in RNFLT (retina nerve fiber layer thickness) before and after the application of pressure (100 mmHg) to the normal eye ball during LASIK or for the experiment for 45 seconds in 2 minutes, one month, 7 month, and 13 month.
9. No confidence interval was reported in the two studies.
10. The internal and external validities were good in the studies for using locally.

Since glaucoma patients are contraindicated for LASIK surgery and it is a rare to see patients having sick optic disc desire laser refractive surgery, there are neither systematic reviews nor randomized control studies available as reference for this case. Furthermore, the alternative laser refractive procedure such as PRK is available for those suspicious cases; most surgeons will choose the alternative way to avoid catastrophic result.

The two experimental studies showed no statistically difference in RNFLT (retina nerve fiber layer thickness) before and after the application of pressure (100 mmHg) to the eye ball during LASIK or for 45 seconds in normal eyes. It revealed the safety of LASIK to normal eyes with the application of 100 mmHg pressure for 45 seconds during surgery by quantitatively measuring the change of nerve fiber thickness using scanning laser tomography or nerve fiber analyzer (laser diagnostic technology).

All the cases that report with abnormal optic nerve showed severe vision decrease due to LASIK. The case series of Lee had two identical conditions of this case (optic disc edema) and suffered severe vision loss after LASIK. However, in Cameron's case with normal optic nerve before operation, there was development of optic neuropathy after LASIK.

## 3. Discussion

The evidence demonstrated that the pressure (100 mmHg pressure for 45 seconds) applies to normal eyes either during LASIK procedure or in experimental study showed no statistically difference in RNFLT. The comparison of the thickness of RNFL before and after the pressure can indicate any loss of nerve fiber in the retina due to the pressure. However, this was only on normal eyes. There were no similar experimental studies on the sick optic disc patients for control. It may be due to the risky nature of the disease that was learned from case series. Therefore, the case reports and case series provided valuable evidences for this paper. Those authors concluded that optic nerve neuropathy can be a potential dangerous complication of LASIK even with normal optic disc before operation. This report may also be relevant for the up coming laser cataract surgery.

It is imperative to carefully examine, every patient's optic nerve with fundus scope before LASIK procedure. If there is any suspicion, scanning laser tomography or nerve fiber analyzer (laser diagnostic technology) should be used to further evaluate the RNFLT. Visual field test may be necessary to compare with nerve fiber analyzer.

This patient's insistence to proceed with laser vision correction despite the abnormal optic disc edema was carefully consulted with evidence in the literature. PRK was recommended to the patient as a safer alternative laser vision correction, since it does not require pressure suction on the eye ball. The drawbacks such as more discomfort than LASIK in the first 24 hours requires more healing time than LASIK, and trauma may cause complications after surgery were explained to patient. 

This patient had PRK and enjoyed 6/6 (20/20) vision in both eye with an uneventful post-op course and the same optic disc appearance as before operation.

## Figures and Tables

**Table 1:** Case reports.

Author	Number of case	Intervention	Outcome measure	Result
Cameron 2001	1	LASIK	Optic neuropathy	↓ vision
Lee 2000	4	LASIK	Optic neuropathy	↓ vision
Montezuma 2008	1	epi-LASIK	Optic neuropathy	↓ vision

**Table 2:** Investigational studies.

Author	Number of cases	Intervention	Outcome measure	Result
Lester 2002	11	100 mmHg 45 sec Compression	RNFLT	No change
Hamada 2006	53	LASIK	RNFLT	No change

Note: RNFLT: retina nerve fiber layer thickness.
